# The Importance of Perceived Procedural Justice Among Defendants With a Non-Western Background Involved in Dutch Criminal Cases

**DOI:** 10.3389/fpsyg.2021.746364

**Published:** 2021-11-29

**Authors:** Lisa F. M. Ansems, Kees van den Bos, Elaine Mak

**Affiliations:** ^1^School of Law, Utrecht University, Utrecht, Netherlands; ^2^Department of Psychology, Utrecht University, Utrecht, Netherlands

**Keywords:** perceived procedural justice, outcome judgments, perceived everyday discrimination, attenuation, reversal, critical test, criminal defendants, external attributions

## Abstract

This study aims to put perceived procedural justice to a critical test in the context of Dutch criminal court hearings. To that end, we surveyed 198 criminal defendants to examine whether their perceptions of procedural fairness were significantly associated with trust in judges and intentions to protest against judicial rulings, among other variables. We also examine the possibility that sometimes *un*fair procedures may have nice aspects, because they offer opportunities to attribute negative outcomes to external causes. Previous studies conducted in different settings support this line of reasoning by showing that associations between perceived procedural justice and other variables are sometimes attenuated or even reversed, particularly when people feel strongly evaluated. The current study takes these insights into the novel context of Dutch criminal court hearings by focusing on defendants with a non-Western ethnic-cultural background. Some of these defendants may feel negatively evaluated by society, which can manifest as a high level of perceived discrimination. Thus, we examine whether the associations between perceived procedural justice and important other variables may be attenuated or reversed depending on respondents’ perceptions of everyday discrimination and their outcome judgments. Our results revealed significant associations between perceived procedural justice on the one hand and trust in judges and protest intentions on the other hand, which remained intact regardless of perceptions of everyday discrimination and outcome judgments. Hence, even in this real-life courtroom context, procedural justice was a relevant concern. Taken together, our findings support the importance of perceived procedural justice, even when it is put to a critical test.

## Introduction

### Procedural Justice and the Fair Process Effect

Fair and just procedures are key aspects of law. Issues of procedural fairness, when viewed from a legal perspective, concern the extent to which legal procedures meet standards laid down in statutes, case law, and unwritten legal principles. In contrast, social psychologists empirically study the extent to which procedures correspond with citizens’ ideas about fairness and justice. These experiences of being treated fairly by decision-making authorities are referred to as perceived procedural justice^1^ (Lind and Tyler, 1988; Tyler and Lind, 1992).

When people perceive procedures as fair, they tend to be more satisfied with the outcomes of their cases and more inclined to accept those outcomes (Thibaut and Walker, 1975; Lind et al., 1993; Van den Bos et al., 2014). They also tend to report higher levels of self-esteem and trust in judges (Koper et al., 1993; Sedikides et al., 2008; Grootelaar, 2018). Other important attitudes and behaviors associated with perceived procedural justice are perceived legitimacy, cooperation with legal authorities, and compliance with the law (Paternoster et al., 1997; Tyler and Huo, 2002; Tyler, 2006). Such favorable responses to perceived procedural justice are generally referred to as the fair process effect (Folger et al., 1979; Van den Bos, 2015).

There are various explanations for people’s concern with issues of procedural fairness (see, e.g., Van den Bos, 2005). For instance, in their pioneering research on this topic, Thibaut and Walker (1975) propose an instrumental explanation of the fair process effect by suggesting that people care about procedural justice because fair procedures are more likely to yield fair and favorable outcomes. Others argue that people care about procedural fairness for relational reasons, as being treated fairly communicates to them that they are valued members of society (e.g., Lind and Tyler, 1988; Tyler and Lind, 1992). Furthermore, information about procedural justice may help to make sense of uncertain situations. For instance, when people do not know whether or not they can trust authorities, they may look to whether or not these authorities treat them fairly and use this information as a heuristic substitute (e.g., Lind et al., 1993; Van den Bos and Lind, 2002).

The current study puts procedural justice and the fair process effect to a critical test by assessing whether the associations between procedural justice perceptions and important other variables hold in the real-life courtroom context of Dutch criminal cases. Contrary to the psychological laboratories which provide the research context of many procedural justice studies, criminal court hearings involve actual stakes, with defendants risking sanctions ranging from fines to community service and imprisonment. In addition, convictions can have serious consequences for defendants’ positions on the job market. Hence, one may wonder whether perceived procedural fairness is a relevant concern for defendants in these criminal cases, as they may be much more concerned with their case outcomes.

In an early critique of procedural justice research, Hayden and Anderson (1979; see also Anderson and Hayden, 1981) point to the use of simulation experiments, which necessarily involve a simplification of social situations, and the resulting limitations as to the conclusions that can be drawn from these studies. Others, too, note the importance of considering real-world complexities. For example, results from a study by Berrey et al. (2012) suggest that litigants involved in employment discrimination cases often did not distinguish between procedures and outcomes, and that they defined fairness in terms of whether or not the procedure benefited their own side. In line with this, Jenness and Calavita (2018) argue that, in their sample of incarcerated men in the United States, participants’ concerns about procedural fairness were largely subordinate to (or even defined by) their outcome concerns.

In contrast, other studies found that perceptions of procedural fairness matter even to people involved in high stakes cases. For instance, Landis and Goodstein (1986) reported that inmates’ perceptions of outcome fairness were associated with both procedural and outcome issues, but that procedural characteristics were dominant in this regard. In addition, Casper et al. (1988) showed that perceived procedural justice was significantly associated with multiple measures of outcome satisfaction among defendants in felony cases. Other findings also indicate the importance of perceived procedural justice in criminal justice contexts (e.g., Tyler, 1984, 1988, 2006; Paternoster et al., 1997; Tyler and Huo, 2002).

To shed further light on this issue, the current paper focuses on the context of real-life criminal court hearings involving actual stakes and examines whether perceptions of procedural fairness are associated with trust in judges and intentions to protest against the judicial ruling, among other variables. More specifically, as our first hypothesis we propose the following:

Defendants with higher levels of perceived procedural justice report more trust in judges, more positive outcome judgments, lower intentions to protest against their outcomes, and higher levels of state self-esteem (i.e., self-esteem at the moment of filling out the questionnaire; Hypothesis 1).

### Attenuated or Reversed Fair Process Effects

Our study also adds to current insights into procedural justice in criminal justice contexts by examining the possibility that sometimes *un*fair procedures may have nice aspects (Van den Bos et al., 1999). As explained by Brockner et al. (2009, p. 185), these “reductions in people’s desire for higher process fairness” may result in attenuated or even reversed fair process effects. That is, the associations between perceived procedural justice and relevant other variables may be weakened, possibly to the extent that they are no longer statistically significant, or reversed, such that people respond more favorably to perceived procedural unfairness than to perceived procedural fairness (Brockner et al., 2009).

One explanation for attenuated or reversed fair process effects is people’s self-enhancement motive. That is, people often want to feel good about themselves (Leary and Terry, 2013). When people perceive procedures as fair, they are more likely to view themselves as personally responsible for their outcomes and thus make more internal attributions (Leung et al., 2001; Brockner et al., 2009). Unfavorable outcomes may then harm their self-esteem (Weiner, 1985). To protect their self-esteem, people may look for opportunities to attribute these negative outcomes to external causes (Cohen 1982). Procedures that people perceive as unfair offer such external attribution opportunities (Brockner et al., 2003). That is, people may maintain their self-esteem by attributing negative outcomes to the perceived unfairness of procedures rather than to themselves. Hence, for people who receive negative outcomes unfair procedures can have nice aspects, at least under some circumstances (Van den Bos et al., 1999).

This line of reasoning is supported by a number of empirical studies, which show that the positive association between perceived procedural justice on the one hand and self-esteem or related measures on the other hand may sometimes be attenuated or even reversed when outcomes are perceived as unfavorable in work contexts (e.g., Gilliland, 1994; Ployhart et al., 1999; Schroth and Shah, 2000; Brockner et al., 2008, 2009; Brockner, 2010). Some studies have found attenuated or reversed fair process effects when examining different kinds of dependent variables. Thus, possibly, people’s reduced threat to sense of self due to perceived procedural unfairness (or, vice versa, their heightened threat to sense of self due to perceived procedural fairness) translates into other kinds of reactions. For instance, one study found an attenuated fair process effect on trust in judges among research participants with relatively high external attribution ratings (see Ansems, 2021). In addition, Holmvall and Bobocel (2008) found a reversed fair process effect on measures of perceived outcome fairness and outcome satisfaction. Furthermore, Van den Bos et al. (1999) found a reversed fair process effect on protest intentions in the face of unfavorable outcomes manipulated in laboratory experiments. In their study, the reversal was triggered by the strength of the evaluative context: Participants who felt strongly evaluated during the decision-making procedure reported lower protest intentions when they perceived the procedure as unfair rather than fair. Thus, feeling strongly evaluated can play an important role in attenuating or reversing the fair process effect.

### Perceived Everyday Discrimination

To examine these issues, the current research focuses on perceived procedural justice among defendants with a non-Western ethnic-cultural background^2^ involved in Dutch criminal cases. Some of these defendants may feel negatively evaluated by society, which can manifest as a high level of perceived discrimination (Huijnk and Andriessen, 2016). We propose that, as a result, these defendants might respond differently to perceptions of procedural fairness during their court hearings (Van den Bos et al., 1999). Hence, we assess whether the associations between perceived procedural justice and relevant other variables may be attenuated or reversed depending on how much discrimination defendants experience in their daily lives.

In doing so, we also take into account research on discrimination which examines processes similar to the self-enhancement processes outlined earlier. Perceived discrimination can lead to various problems, including stress and reduced psychological well-being (Major et al., 2002). At the same time, experiencing discrimination may enable people to maintain their self-esteem, as it reduces their sense of personal responsibility and deservingness of negative outcomes (Crocker and Major, 1989; Major, 1994). Hence, attributing negative events to discrimination rather than one’s personal qualities is a coping strategy people can use to counter the negative impact these events may otherwise have on their self-esteem (Major et al., 2002). Building on these insights, we formulate our second hypothesis as follows:

There is a two-way interaction between perceived procedural justice and perceived everyday discrimination, such that defendants who experience relatively high levels of everyday discrimination show attenuated or reversed associations between perceived procedural justice and our other variables (i.e., trust in judges, outcome judgments, protest intentions, and state self-esteem; Hypothesis 2).

### Outcome Judgments

In addition to perceptions of everyday discrimination, we examine the potentially moderating role of defendants’ outcome judgments (i.e., how positively or negatively they judge their outcomes). According to Brockner and Wiesenfeld (1996), receiving negative outcomes triggers sense-making processes. As a result, people may pay more attention to issues of procedural fairness. Thus, the fair process effect may be strengthened when people receive negative outcomes. At the same time, people may look for opportunities to attribute these negative outcomes to external causes in order to protect their self-esteem (Cohen 1982), as explained earlier. Because procedures that are perceived as unfair offer such opportunities, the fair process effect may be attenuated or reversed (Brockner et al., 2009).

Combining these possibilities, our third hypothesis suggests that the associations between perceived procedural justice and our other variables may be moderated by defendants’ outcome judgments. When defendants judge their outcomes more negatively, this may either strengthen the associations between perceived procedural justice and our other variables (Brockner and Wiesenfeld, 1996) or, alternatively, attenuate or even reverse these associations (Brockner et al., 2009). Thus, we formulate our third hypothesis as follows:

There is a two-way interaction between perceived procedural justice and outcome judgments, such that defendants who judge their outcomes more negatively show stronger, attenuated, or reversed associations between perceived procedural justice and our other variables (i.e., trust in judges, protest intentions, and state self-esteem; Hypothesis 3).^3^

### Research Context

To study these issues, we conducted a face-to-face survey among 198 defendants with a non-Western ethnic-cultural background involved in Dutch single judge criminal cases. In the Dutch court system, single judges handle relatively simple criminal cases in which the sanction demanded by the public prosecutor does not exceed 1year of imprisonment. Typical cases handled by single judges include theft, simple assault, and traffic offenses such as driving under the influence. Defendants can be represented by a lawyer, or they can choose to defend themselves. Usually, single judge criminal court hearings last around 30min and judges render a verdict directly afterward. The description below provides more details on the Dutch legal context and some of the main differences with the legal system of (for example) the United States.

“First, Dutch criminal proceedings take place largely “on paper”. That is, the emphasis is on the pretrial investigation rather than on court hearings, which generally last around 30min in small criminal cases and 60–90min in more severe ones. Second, the Dutch legal system does not have a plea-bargaining system like the United States. Third, the administration of justice is entirely in the hands of professional judges; the Dutch legal system does not have bifurcated proceedings in which defendants’ guilt is determined by a jury and their sentences by a judge. Fourth, criminal court hearings in the Netherlands are less adversarial than in the United States. That is, Dutch hearings involve an active role for judges and traditionally treat defendants as subject of the investigation, whereas the United States legal system involves more passive judges and views the court hearing as a clash of parties.” (Ansems et al., 2020, p. 648)

Relatively, many defendants in Dutch criminal cases have a non-Western ethnic-cultural background. People with a Moroccan or Antillean background in particular are overrepresented in Dutch crime statistics, which could be due partly to negative stereotypes and ethnic profiling (Huijnk and Andriessen, 2016). Discrimination is a relevant issue in Dutch society more broadly, too, as several studies show that people with a non-Western migration background report relatively high levels of perceived discrimination (Huijnk et al., 2015; Huijnk and Andriessen, 2016; Andriessen et al., 2020). Indeed, there are signs that Dutch people with a migration background may be discriminated in important life domains (e.g., Thijssen et al., 2019).

Against this backdrop, the present study examines whether experiences of everyday discrimination and outcome judgments may moderate how defendants with a non-Western ethnic-cultural background involved in Dutch criminal cases react to perceived procedural justice during their court hearings. Our study helps to refine current insights into perceived procedural justice by focusing not only on the possible robustness of associations between perceived procedural justice and relevant other variables, but also on the potential attenuation or reversal of these associations. In doing so, we take insights from previous studies conducted in work settings and the psychological laboratory (e.g., Van den Bos et al., 1999; Brockner et al., 2009) and apply them to the novel context of Dutch criminal cases.

In addition, because of our focus on defendants with a non-Western ethnic-cultural background, this study sheds light on experiences of a relatively underinvestigated research population. Contrary to research participants in many other procedural justice studies, respondents in the current study generally have non-WEIRD (Western, Educated, Industrialized, Rich, and Democratic; Henrich et al., 2010) backgrounds. Taken together, we conducted our study in a real-life courtroom context, focusing on defendants with diverse ethnic-cultural backgrounds who might respond differently to perceived procedural justice. In this way, we critically examine the role of perceived procedural justice in Dutch criminal court hearings.

## Materials and Methods

### Sample

Our sample consisted of 198 defendants with a non-Western ethnic-cultural background who appeared before a single judge at the court of the Mid-Netherlands in Utrecht, Lelystad, and Almere.^4^ Table 1 details sample characteristics.

**Table 1:** Sample description.

Categorical variables			
Variable	Category	*N*	%
Location of court hearing	Utrecht	190	96.0
	Lelystad	6	3.0
	Almere	2	1.0
Gender	Male	178	90.4
	Female	19	9.6
Highest completed level of education	Primary school	14	7.4
	Secondary school	81	42.6
	Secondary vocational education	62	32.6
	Higher professional education	24	12.6
	University	6	3.2
	Other	3	1.6
	*Special needs education*	*1*	*0.5*
	*None at all*	*2*	*1.1*
Ethnic-cultural background	Moroccan	85	42.9
	Surinam	25	12.6
	Turkish	20	10.1
	Antillean	12	6.1
	Other (e.g., Somalian, Iraqi, Afghan)	58	29.3
Offense	Assault or violence	57	30.0
	Theft, embezzlement, fencing, or breaking and entering	45	23.7
	Traffic offense (e.g., driving under the influence)	43	22.6
	Threatening someone	19	10.0
	Drug offense	17	8.9
	Insulting someone	13	6.8
	Destruction	12	6.3
	Scam or fraud	5	2.6
Case outcome	Convicted with imposition of sanction or measure (conditional or unconditional)	152	79.2
	*Community service*	*108*	*65.1*
	*Fine*	*51*	*30.7*
	*Prison sentence*	*27*	*16.3*
	Acquitted	26	13.5
	Found guilty without imposition of sanction or measure	13	6.8
	Discharged from further prosecution	2	1.0
Legal assistance	By lawyer	136	70.1
	By someone else	5	2.6
	None	53	27.3
Number of previous criminal court hearings	None	69	35.0
	One	48	24.4
	Two to ten	66	33.5
	More than ten	14	7.1
**Continuous variables**			
**Variable**	**Range (years)**	**Average (years)**	***SD***
Age	18–66	30.10	10.75

### Research Procedure

After gaining the court’s permission to conduct the study, we collected our data between January 21 and October 15, 2019. Except for the summer break, the first author went to the court almost every work day during this period to collect data and stayed there for the duration of the criminal court’s session that day (most often from 9 to 18h, sometimes from 9 to 13h, or from 13 to 18h). Among the causes for the relatively long duration of data collection were our focus on defendants with a non-Western ethnic-cultural background, the fact that many defendants did not appear for their court hearings, and some defendants’ poor command of Dutch. Defendants appeared before the court of the mid-Netherlands if they were accused of a crime that had been committed in that geographic region or if they were living there.

The first author approached defendants in the court hallway after they had made their presence known at the counter to ask whether they were willing to participate in a study about how fairly and justly they felt they were treated during their court hearings, indicating that they would be thanked for their participation with a small token of appreciation. Seventeen respondents (8.6% of the sample) were approached by a research assistant. We approached respondents before the start of their court hearings as much as possible to ask whether they were willing to participate in the study once their court hearings had ended. When it was not possible to approach respondents before the start of their court hearings – for instance, because they appeared for their hearings only very last minute or because they were consulting with their lawyers – respondents were approached immediately after their court hearings.

Our study procedures were approved by the ethical board of the Faculty of Law, Economics, Governance, and Organization at Utrecht University. Following these approved procedures, and because our study focused on how people with non-Western ethnic-cultural backgrounds would respond to issues of procedural justice, we approached defendants who appeared to have a non-Western ethnic-cultural background for participation in our study. Therefore, based on their names and physical appearance, we made an initial assessment whether people appeared to have a non-Western ethnic-cultural background and invited those people to take part in our study.^5^ At the start of the questionnaire, we informed respondents that we were interested in how people who were born in a different country than the Netherlands and people whose parents or other ancestors were born in a different country than the Netherlands would evaluate how they were treated during their court hearings, their trust in Dutch judges, and how they felt treated in their daily lives, among other things. We ensured that we always treated people respectfully throughout the entire study. In fact, while filling out the questionnaire or afterward, multiple respondents indicated that they appreciated studies like ours, as these are needed to help to understand discrimination in Dutch society.

We also note that, due to our way of sampling, we may have missed people who were eligible for participation in our study but whose physical appearance or name was not clearly non-Western. Although we cannot rule out that this may have affected our results, we do not think this was a big problem in the current study. After all, these people may be less likely to feel discriminated against based on their ethnic-cultural background, whereas we were particularly interested in defendants who experience relatively high levels of discrimination in Dutch society and might therefore respond differently to perceived procedural justice. Our impression is that we were successful in conducting our study in responsible and sound ways. In the Discussion section, we note limitations of our study that may inspire future research.

In addition to having a non-Western ethnic-cultural background, our other inclusion criteria were that defendants had received the outcome of their case and that they had a sufficient command of Dutch. We immediately filtered out respondents who, when starting to fill out the questionnaire in the court hallway, turned out not to meet our inclusion criteria and thus turned out to be ineligible for participation in our study. In total, we approached 447 defendants (excluding defendants who, based on this initial screening, turned out to be ineligible for participation). Of those 447 defendants, 210 filled out our questionnaire, resulting in a response rate of 47.0%. In a later stage, before conducting our analyses, we filtered out the questionnaires that did not indicate the respondent’s ethnic-cultural background or that had a very large number of missing values. Thus, the final sample consisted of 198 respondents. A power analysis (Faul et al., 2007) showed that, to achieve sufficient statistical power of 0.80 (Cohen et al., 2003) to detect the two-way interaction between perceived everyday discrimination and perceived procedural justice, with *α*=0.05 and a relatively small effect size (*f*^2^=0.04), at least 191 respondents were needed.

Most respondents completed the questionnaire directly. Six respondents (3.0% of the sample) filled it out at home and sent it to us in an envelope with prepaid postage stamps. The respondents who filled out the questionnaire directly often did so themselves, while 25 respondents (12.8% of the sample) preferred having the questions read out loud by the researcher. Before respondents filled out the questionnaire, we explained that the research focused on persons who were born in a different country and persons whose parents or other ancestors were born in a different country. In addition, we told respondents that participation was voluntary and anonymous, and that the research was conducted independently of the court and the Public Prosecution Service.

After they completed the questionnaire, we thanked respondents for their participation and offered to send them a summary of our research results, which we sent to interested respondents later. During the entire period of data collection, we kept an extensive logbook detailing relevant background information to the research, such as information obtained through informal conversations with defendants and defense lawyers.

### Measures

Our main variables were perceived procedural justice, outcome judgments, perceived everyday discrimination, trust in Dutch judges, protest intentions, and state self-esteem. The questionnaire started with those variables relating to the court hearing (perceived procedural justice, outcome judgments, protest intentions, and trust in Dutch judges) and then assessed variables targeting respondents’ perceptions more generally (state self-esteem and perceived everyday discrimination).^6^

We measured perceived procedural justice with a six-item scale based on the findings of a recent qualitative interview study conducted in the same criminal courtroom context as the current study (Ansems et al., 2020). Our survey items corresponded with the six core components of perceived procedural justice among defendants as revealed by the interview study. Specifically, we asked respondents to indicate, on a Likert scale from 1 (*totally disagree*) to 7 (*totally agree*), to what extent they agreed with the following six statements: “During the court hearing, I was treated in a pleasant way,” “During the court hearing, I was treated in an unprejudiced manner,” “During the court hearing, I was sufficiently able to tell my side of the story,” “During the court hearing, my side of the story was listened to,” “During the court hearing, everything important has been taken into account,” and “During the court hearing, my case was treated in a careful manner.” Together, these items formed a reliable scale (*α*=0.82) on which higher scores reflect higher levels of perceived procedural justice. Therefore, we report the results of our analyses without the additional 11 items which we included as backup in case the six-item scale would turn out to be unreliable and which were based on previous work in other courtroom settings (Grootelaar and van den Bos, 2018).^7^

We also assessed respondents’ outcome judgments, which in this study include outcome satisfaction, perceived outcome fairness, and perceived outcome favorability. Our outcome judgments scale was largely based on previous research in a similar context (Grootelaar and van den Bos, 2018) and consisted of six items: “I find this ruling fair,” “I find this ruling favorable,” “I am satisfied with the judge’s ruling,” “I find this ruling just,” “The judge’s ruling has positive consequences for me,” and “I agree with the judge’s ruling.” Again, respondents indicated on a scale from 1 to 7 to what extent they agreed with these statements, and for each respondent, we took the average of their scores on these items to calculate their scores on our outcome judgments scale (*α*=0.97). Higher scores on this scale indicate that respondents judged their outcomes more positively.

We examined perceived everyday discrimination with the 10-item version of the everyday discrimination scale (Williams et al., 1997, 2008). We asked respondents to indicate on a scale from 1 (*never*) to 6 (*almost every day*) how often they encountered the following events in their daily lives: “In my day-to-day life, I am treated with less courtesy than other people are,” “In my day-to-day life, I am treated with less respect than other people are,” “In my day-to-day life, I receive poorer service than other people at restaurants or stores,” “In my day-to-day life, people act as if they think I am not smart,” “In my day-to-day life, people act as if they are afraid of me,” “In my day-to-day life, people act as if they think I am dishonest,” “In my day-to-day life, people act as if they are better than I am,” “In my day-to-day life, I am called names or insulted,” “In my day-to-day life, I am threatened or harassed,” and “In my day-to-day life, I am followed around in stores.” Together, these items formed a reliable perceived everyday discrimination scale (*α*=0.91). Higher scores on this scale reflect higher levels of perceived everyday discrimination. In addition, respondents who answered “a few times a year” (score 3 on the six-point scale) or more often to at least one question were asked to indicate what they thought was the main reason for these experiences: their gender, their age, their religion, their ethnic-cultural background, their level of education, their level of income, and/or some other reason (which they could then write down). In this way, we assessed perceived grounds of discrimination.

We solicited their trust in Dutch judges with items that target this construct in a way that we deemed as direct and straightforward as possible (see also Grootelaar and van den Bos, 2018). Specifically, we asked respondents to indicate on a scale from 1 (*totally disagree*) to 7 (*totally agree*) to what extent they agreed with the following five statements: “I have faith in Dutch judges,” “I deem Dutch judges trustworthy,” “I trust Dutch judges,” “I do not trust Dutch judges” (reverse coded), and “I feel like Dutch judges cannot be trusted” (reverse coded). Respondents’ answers on these items were averaged into a reliable trust in Dutch judges scale (*α*=0.90) on which higher scores reflect higher levels of trust. We also included an additional sixth item asking respondents to express their trust in Dutch judges with a grade between 1 (*lowest*) and 10 (*highest*), in line with the grading system used in Dutch schools.

Following Stahl et al. (2008), we assessed protest intentions by asking respondents to indicate on a scale from 1 (*not at all*) to 7 (*very much*) to what extent they would like to criticize the ruling and to what extent they would like to protest against the ruling. Respondents’ answers on these two items were averaged into a reliable protest intentions scale (*α*=0.85). Higher scores on this scale represent stronger protest intentions.

Finally, to measure respondents’ state self-esteem at the moment they filled out our questionnaire we adapted the global self-esteem scale of Rosenberg (1965) to measure state global self-esteem. Hence, respondents were asked to indicate on a scale from 1 (*totally disagree*) to 7 (*totally agree*) to what extent they agreed with the following 10 statements: “Now, at this moment, I am satisfied with myself,” “Now, at this moment, I think I am no good at all” (reverse coded), “Now, at this moment, I feel that I have a number of good qualities,” “Now, at this moment, I am able to do things as well as most other people,” “Now, at this moment, I feel like I do not have much to be proud of” (reverse coded), “Now, at this moment, I feel useless” (reverse coded), “Now, at this moment, I feel that I am a person of worth, at least on an equal plane with others,” “Now, at this moment, I wish I could have more respect for myself” (reverse coded), “Now, at this moment, I feel like I am a failure” (reverse coded), and “Now, at this moment, I take a positive attitude toward myself.” Respondents’ answers on these items were averaged into a reliable state self-esteem scale (*α*=0.83) on which higher scores reflect higher state self-esteem.

We also assessed relevant background variables, asking respondents to indicate whether they had legal assistance during their court hearings, their number of previous court hearings before a criminal judge, their highest completed level of education, their gender, and their age. At the end of the questionnaire, respondents could write down remarks or issues they deemed important that had not been the subject of our questions.^8^

## Results

### Descriptive Statistics and Bivariate Correlations

All statistical analyses were conducted with IBM SPSS software. Table 2 presents means, standard deviations, and bivariate correlations for our main variables and background variables.

**Table 2:** Means, standard deviations, and correlations for the main variables and background variables.

Variable	*M*	SD	1	2	3	4	5	6	7	8	9	10	11	12
1. Procedural justice	5.38	1.27	–											
2. Outcome judgments	4.55	2.18	0.59^***^	–										
3. Discrimination	2.48	1.16	−0.06	−0.12	–									
4. Protest intentions	3.31	2.02	−0.45^***^	−0.60^***^	0.26^***^	–								
5. Trust in judges	5.09	1.55	0.50^***^	0.42^***^	−0.28^***^	−0.34^***^	–							
6. Trust in judges grade	6.83	2.06	0.47^***^	0.40^***^	−0.22^**^	−0.34^***^	0.79^***^	–						
7. Self–esteem	5.62	1.09	0.19^**^	0.16^*^	−0.22^**^	−0.28^***^	0.26^***^	0.15^*^	–					
8. Legal assistance (0=*no*, 1=*yes*)	–	–	−0.19^**^	−0.06	0.07	0.05	−0.14	−0.17^*^	0.00	–				
9. Previous hearings	2.14	1.00	−0.06	0.02	0.11	−0.03	−0.15^*^	−0.18^*^	−0.13	0.11	–			
10. Level of education	5.37	2.26	−0.05	−0.11	−0.08	0.05	0.02	0.02	−0.01	−0.01	−0.33^***^	–		
11. Gender (0=*female*, 1=*male*)	–	–	−0.03	−0.11	0.05	0.06	−0.05	0.03	−0.04	−0.09	0.17	−0.02	–	
12. Age	30.10	10.75	0.10	0.10	−0.10	−0.15^*^	0.17^*^	0.14	0.02	−0.14	0.11	−0.19^*^	−0.06	–

*The number of previous court hearings was measured on a five-point scale (1=0, 2=1, 3=2–10, 4=11–20, 5=more than 20). Highest completed level of education was measured on a nine-point scale, ranging from primary school (coded as 1) to university (coded as 9). Perceived grounds for discrimination included respondents’ ethnic-cultural background (N=93; 63.3% of the sample), religion (N=56; 38.1% of the sample), gender (N=25; 17.0% of the sample), age (N=21; 14.3% of the sample), level of income (N=17; 11.6% of the sample), and level of education (N=17; 11.6% of the sample)*.

* *p<0.05*;

** *p<0.01*;

*** *p<0.001*.

As shown in Table 2, there were statistically significant relationships between some of our background variables (i.e., legal assistance, number of previous court hearings, and age) and some of our main variables (i.e., perceived procedural justice, trust in judges, and protest intentions). Hence, we controlled for legal assistance, number of previous court hearings, and age in the hierarchical regression analyses reported below by entering them in Step 1 of the analysis. Main effects were entered in Step 2, and two-way interactions were entered in Step 3. Following recommendations by Cohen et al. (2003), all continuous independent variables (including quasi-interval variables) were standardized before being entered into the equation when the equation involved an interaction effect. When reporting the results of these hierarchical regression analyses, we focus on the last step in the analysis that significantly added to the amount of explained variance in the dependent variables in our regression equations.

### Reacting to Procedural Justice

Hypothesis 1 predicted that defendants with higher levels of perceived procedural justice report more trust in judges, more positive outcome judgments, lower intentions to protest against their outcomes, and higher levels of state self-esteem. This hypothesis was supported by our results. That is, respondents who felt treated more fairly during their court hearings showed more trust in judges (*β*=0.50) and gave their trust in judges higher grades (*β*=0.44), judged their outcomes more positively (*β*=0.59), indicated lower protest intentions (*β*=−0.45), and reported higher state self-esteem (*β*=0.22). Further details are presented in Tables 3–6.

**Table 3:** Trust in judges regressed on procedural justice and relevant background variables.

Variable	Step 1				95% CI for *b*	Step 2				95% CI for *b*
	*b*	*β*	*t*	*p*		*b*	*β*	*t*	*p*	
Legal assistance (0=*no*, 1=*yes*)	−0.37	−0.11	−1.48	0.141	−0.86, 0.12	−0.08	−0.02	−0.36	0.718	−0.51, 0.35
Previous hearings	−0.21	−0.14	−1.91	0.058	−0.43, 0.01	−0.18	−0.12	−1.86	0.065	−0.36, 0.01
Age	0.03	0.18	2.50	0.013	0.01, 0.05	0.02	0.15	2.44	0.016	0.00, 0.04
Procedural justice						0.61	0.50	7.98	0.000	0.46, 0.76
*df*	3					4				
*F*	4.31, *p* =0.006					20.23, *p* =0.000				
*F* change	4.31, *p* =0.006					63.62, *p* =0.000				
*R^2^*	0.07					0.31				
Adjusted *R*^2^	0.05					0.29				
*N*	189					189				

*The number of previous court hearings was measured on a five-point scale (1=0, 2=1, 3=2–10, 4=11–20, 5=more than 20)*.

**Table 4:** Outcome judgments regressed on procedural justice and relevant background variables.

Variable	Step 1				95% CI for *b*	Step 2				95% CI for *b*
	*b*	*β*	*t*	*p*		*b*	*β*	*t*	*p*	
Legal assistance (0=*no*, 1=*yes*)	−0.27	−0.06	−0.75	0.455	−0.98, 0.44	0.21	0.04	0.72	0.475	−0.38, 0.80
Previous hearings	0.05	0.02	0.33	0.745	−0.26, 0.37	0.11	0.05	0.83	0.406	−0.15, 0.37
Age	0.02	0.08	1.08	0.282	−0.01, 0.05	0.01	0.05	0.77	0.442	−0.02, 0.03
Procedural justice						1.03	0.59	9.69	0.000	0.82, 1.24
*df*	3					4				
*F*	0.72, *p* =0.541					24.27, *p* =0.000				
*F* change	0.72, *p* =0.541					93.85, *p* =0.000				
*R* ^2^	0.01					0.34				
Adjusted *R*^2^	−0.00					0.33				
*N*	190					190				

*The number of previous court hearings was measured on a five-point scale (1=0, 2=1, 3=2–10, 4=11–20, 5=more than 20)*.

**Table 5:** Protest intentions regressed on procedural justice and relevant background variables.

Variable	Step 1				95% CI for *b*	Step 2				95% CI for *b*
	*b*	*β*	*t*	*p*		*b*	*β*	*t*	*p*	
Legal assistance (0=*no*, 1=*yes*)	0.16	0.04	0.48	0.630	−0.50, 0.82	−0.18	−0.04	−0.60	0.550	−0.78, 0.42
Previous hearings	−0.03	−0.02	−0.20	0.842	−0.32, 0.26	−0.07	−0.04	−0.52	0.601	−0.33, 0.19
Age	−0.02	−0.13	−1.71	0.088	−0.05, 0.00	−0.02	−0.10	−1.52	0.130	−0.04, 0.01
Procedural justice						−0.72	−0.45	−6.74	0.000	−0.94, −0.51
*df*	3					4				
*F*	1.21, *p* =0.308					12.49, *p* =0.000				
*F* change	1.21, *p* =0.308					45.46, *p* =0.000				
*R* ^2^	0.02					0.21				
Adjusted *R*^2^	0.00					0.20				
N	190					190				

*The number of previous court hearings was measured on a five-point scale (1=0, 2=1, 3=2–10, 4=11–20, 5=more than 20)*.

**Table 6:** Self-esteem regressed on procedural justice and relevant background variables.

Variable	Step 1				95% CI for *b*	Step 2				95% CI for *b*
	*b*	*β*	*t*	*p*		*b*	*β*	*t*	*p*	
Legal assistance (0=*no*, 1=*yes*)	0.05	0.02	0.26	0.793	−0.31, 0.40	0.14	0.06	0.77	0.440	−0.21, 0.49
Previous hearings	−0.14	−0.13	−1.78	0.077	−0.30, 0.02	−0.13	−0.12	−1.68	0.094	−0.28, 0.02
Age	0.00	0.03	0.39	0.696	−0.01, 0.02	0.00	0.02	0.23	0.821	−0.01, 0.02
Procedural justice						0.19	0.22	3.07	0.002	0.07, 0.32
*df*	3					4				
*F*	1.06, *p* =0.366					3.19, *p* =0.015				
*F* change	1.06, *p* =0.366					9.41, *p* =0.002				
*R* ^2^	0.02					0.07				
Adjusted *R*^2^	0.00					0.04				
*N*	189					189				

*The number of previous court hearings was measured on a five-point scale (1=0, 2=1, 3=2–10, 4=11–20, 5=more than 20)*.

### Adding Perceived Everyday Discrimination

Hypothesis 2 proposed that there is a two-way interaction between perceived procedural justice and perceived everyday discrimination, such that defendants who experience relatively high levels of everyday discrimination show attenuated or reversed associations between perceived procedural justice and our other variables (i.e., trust in judges, outcome judgments, protest intentions, and state self-esteem). This hypothesis was not supported by our results, as we did not find significant interaction effects between perceived everyday discrimination and perceived procedural justice. We did find significant main effects of perceived procedural justice, sometimes in addition to significant main effects of perceived everyday discrimination. That is, perceived procedural justice was positively associated with respondents’ trust in judges (*β*=0.48) and the grades they gave their trust in judges (*β*=0.42), their outcome judgments (*β*=0.58), and their state self-esteem (*β*=0.21) and was negatively related to respondents’ protest intentions (*β*=−0.43). In addition, perceived everyday discrimination was negatively associated with trust in judges (*β*=−0.22) and the grades respondents gave their trust in judges (*β*=−0.16) as well as respondents’ state self-esteem (*β*=−0.17) and was positively associated with protest intentions (*β*=0.24). Further details are shown in Tables 7–10.

**Table 7:** Regression results for procedural justice, discrimination, and their interaction on trust in judges.

Variable	Step 1				95% CI for *b*	Step 2				95% CI for *b*	Step 3				95% CI for *b*
	*b*	*β*	*t*	*p*		*b*	*β*	*t*	*p*		*b*	*β*	*t*	*p*	
Legal assistance (0=*no*, 1=*yes*)	−0.37	−0.11	−1.48	0.141	−0.86, 0.12	−0.05	−0.02	−0.25	0.804	−0.47, 0.36	−0.04	−0.01	−0.18	0.858	−0.45, 0.38
Previous hearings	−0.21	−0.14	−1.91	0.058	−0.43, 0.01	−0.14	−0.09	−1.54	0.125	−0.33, 0.04	−0.14	−0.09	−1.46	0.146	−0.32, 0.05
Age	0.28	0.18	2.50	0.013	0.06, 0.50	0.20	0.13	2.11	0.036	0.01, 0.38	0.19	0.13	2.05	0.042	0.01, 0.38
Procedural justice						0.75	0.48	7.94	0.000	0.57, 0.94	0.76	0.49	8.00	0.000	0.57, 0.95
Discrimination						−0.33	−0.22	−3.56	0.000	−0.51, −0.15	−0.35	−0.23	−3.71	0.000	−0.53, −0.16
Discrimination×Procedural justice											0.11	0.07	1.14	0.258	−0.08, 0.30
*df*	3					5					6				
*F*	4.31, *p* =0.006					19.75, *p* =0.000					16.70, *p* =0.000				
*F* change	4.31, *p* =0.006					40.17, *p* =0.000					1.29, *p* =0.258				
*R* ^2^	0.07					0.35					0.36				
Adjusted *R*^2^	0.05					0.33					0.33				
*N*	189					189					189				

*The number of previous court hearings was measured on a five-point scale (1=0, 2=1, 3=2–10, 4=11–20, 5=more than 20)*.

**Table 8:** Regression results for procedural justice, discrimination, and their interaction on outcome judgments.

Variable	Step 1				95% CI for *b*	Step 2				95% CI for *b*	Step 3				95% CI for *b*
	*b*	*β*	*t*	*p*		*b*	*β*	*t*	*p*		*b*	*β*	*t*	*p*	
Legal assistance (0=*no*, 1=*yes*)	−0.27	−0.06	−0.75	0.455	−0.98, 0.44	0.23	0.05	0.78	0.438	−0.36, 0.82	0.24	0.05	0.79	0.430	−0.35, 0.83
Previous hearings	0.05	0.02	0.33	0.745	−0.26, 0.37	0.13	0.06	0.99	0.322	−0.13, 0.39	0.13	0.06	1.01	0.315	−0.13, 0.39
Age	0.17	0.08	1.08	0.282	−0.14, 0.49	0.08	0.04	0.60	0.552	−0.18, 0.34	0.08	0.04	0.58	0.565	−0.19, 0.34
Procedural justice						1.29	0.58	9.58	0.000	1.02, 1.55	1.29	0.58	9.56	0.000	1.02, 1.55
Discrimination						−0.20	−0.09	−1.53	0.127	−0.46, 0.06	−0.21	−0.10	−1.56	0.122	−0.47, 0.06
Discrimination×Procedural Justice											0.04	0.02	0.29	0.774	−0.23, 0.31
*df*	3					5					6				
*F*	0.72, *p* =0.541					20.03, *p* =0.000					16.62, *p* =0.000				
*F* change	0.72, *p* =0.541					48.44, *p* =0.000					0.08, *p* =0.774				
*R* ^2^	0.01					0.35					0.35				
Adjusted *R*^2^	−0.00					0.34					0.33				
*N*	190					190					190				

*The number of previous court hearings was measured on a five-point scale (1=0, 2=1, 3=2–10, 4=11–20, 5=more than 20)*.

**Table 9:** Regression results for procedural justice, discrimination, and their interaction on protest intentions.

Variable	Step 1				95% CI for *b*	Step 2				95% CI for *b*	Step 3				95% CI for *b*
	*b*	*β*	*t*	*p*		*b*	*β*	*t*	*p*		*b*	*β*	*t*	*p*	
Legal assistance (0=*no*, 1=*yes*)	0.16	0.04	0.48	0.630	−0.50, 0.82	−0.22	−0.05	−0.76	0.447	−0.80, 0.36	−0.24	−0.05	−0.82	0.414	−0.82, 0.34
Previous hearings	−0.03	−0.02	−0.20	0.842	−0.32, 0.26	−0.12	−0.06	−0.93	0.354	−0.38, 0.14	−0.13	−0.06	−0.99	0.323	−0.38, 0.13
Age	−0.25	−0.13	−1.71	0.088	−0.55, 0.04	−0.15	−0.07	−1.15	0.253	−0.41, 0.11	−0.14	−0.07	−1.09	0.277	−0.40, 0.12
Procedural justice						−0.88	−0.43	−6.68	0.000	−1.14, −0.62	−0.89	−0.44	−6.73	0.000	−1.15, −0.63
Discrimination						0.48	0.24	3.71	0.000	0.22, 0.73	0.50	0.25	3.82	0.000	0.24, 0.76
Discrimination×Procedural justice											−0.13	−0.06	−0.98	0.328	−0.40, 0.13
*df*	3					5					6				
*F*	1.21, *p* =0.308					13.43, *p* =0.000					11.35, *p* =0.000				
*F* change	1.21, *p* =0.308					31.17, *p* =0.000					0.96, *p* =0.328				
*R* ^2^	0.02					0.27					0.27				
Adjusted *R*^2^	0.00					0.25					0.25				
*N*	190					190					190				

*The number of previous court hearings was measured on a five-point scale (1=0, 2=1, 3=2–10, 4=11–20, 5=more than 20)*.

**Table 10:** Regression results for procedural justice, discrimination, and their interaction on self-esteem.

Variable	Step 1				95% CI for *b*	Step 2				95% CI for *b*	Step 3				95% CI for *b*
	*b*	*β*	*t*	*p*		*b*	*β*	*t*	*p*		*b*	*β*	*t*	*p*	
Legal assistance (0=*no*, 1=*yes*)	0.05	0.02	0.26	0.793	−0.31, 0.40	0.15	0.06	0.87	0.386	−0.19, 0.50	0.16	0.07	0.91	0.365	−0.19, 0.51
Previous hearings	−0.14	−0.13	−1.78	0.077	−0.30, 0.02	−0.11	−0.10	−1.45	0.149	−0.26, 0.04	−0.11	−0.10	−1.40	0.164	−0.26, 0.05
Age	0.03	0.03	0.39	0.696	−0.13, 0.19	−0.00	−0.00	−0.04	0.971	−0.16, 0.15	−0.01	−0.01	−0.08	0.940	−0.16, 0.15
Procedural justice						0.23	0.21	2.92	0.004	0.08, 0.39	0.23	0.21	2.95	0.004	0.08, 0.39
Discrimination						−0.19	−0.17	−2.43	0.016	−0.34, −0.04	−0.20	−0.18	−2.51	0.013	−0.35, −0.04
Discrimination×Procedural justice											0.05	0.05	0.67	0.502	−0.10, 0.21
*df*	3					5					6				
*F*	1.06, *p* =0.366					3.80, *p* =0.003					3.23, *p* =0.005				
*F* change	1.06, *p* =0.366					7.78, *p* =0.001					0.45, *p* =0.502				
*R* ^2^	0.02					0.09					0.10				
Adjusted *R*^2^	0.00					0.07					0.07				
*N*	189					189					189				

*The number of previous court hearings was measured on a five-point scale (1=0, 2=1, 3=2–10, 4=11–20, 5=more than 20)*.

### Adding Outcome Judgments

Hypothesis 3 suggested that there is a two-way interaction between perceived procedural justice and outcome judgments, such that defendants who judge their outcomes more negatively show stronger, attenuated, or reversed associations between perceived procedural justice and our other variables (i.e., trust in judges, protest intentions, and state self-esteem). This hypothesis was not supported by the results, as our analyses did not reveal significant interaction effects between outcome judgments and perceived procedural justice. Our analyses did yield significant main effects of perceived procedural justice, sometimes in addition to significant main effects of outcome judgments. More specifically, we found a positive association between perceived procedural justice and trust in judges (*β*=0.37) and the grade respondents gave their trust in judges (*β*=0.32), a marginally significant association between perceived procedural justice and self-esteem (*β*=0.18), and a negative association between perceived procedural justice and protest intentions (*β*=−0.15). We also found a positive association between outcome judgments and trust in judges (*β*=0.22) and the grade respondents gave their trust in judges (*β*=0.19) and a negative association between outcome judgments and protest intentions (*β*=−0.50). Entering the entire 17-item perceived procedural justice scale into the regression equation rather than the six-item scale yielded the same (non-significant) result regarding the interaction between outcome judgments and perceived procedural justice, the only difference being that the association between outcome judgments and trust in judges was no longer statistically significant. Tables 11–13 present further details.

**Table 11:** Regression results for procedural justice, outcome judgments, and their interaction on trust in judges.

Variable	Step 1				95% CI for *b*	Step 2				95% CI for *b*	Step 3				95% CI for *b*
	*b*	*β*	*t*	*p*		*b*	*β*	*t*	*p*		*b*	*β*	*t*	*p*	
Legal assistance (0=*no*, 1=*yes*)	−0.37	−0.11	−1.48	0.141	−0.86, 0.12	−0.12	−0.03	−0.55	0.585	−0.54, 0.30	−0.12	−0.04	−0.56	0.579	−0.54, 0.31
Previous hearings	−0.21	−0.14	−1.91	0.058	−0.43, 0.01	−0.20	−0.13	−2.09	0.038	−0.38, −0.01	−0.20	−0.13	−2.08	0.039	−0.38, −0.01
Age	0.28	0.18	2.50	0.013	0.06, 0.50	0.22	0.14	2.34	0.020	0.04, 0.41	0.22	0.15	2.33	0.021	0.03, 0.41
Procedural justice						0.58	0.37	4.91	0.000	0.34, 0.81	0.57	0.37	4.75	0.000	0.33, 0.81
Outcome judgments						0.34	0.22	2.94	0.004	0.11, 0.56	0.34	0.22	2.94	0.004	0.11, 0.56
Outcome judgments×Procedural justice											−0.02	−0.01	−0.14	0.888	−0.23, 0.20
*df*	3					5					6				
*F*	4.31, *p* =0.006					18.59, *p* =0.000					15.42, *p* =0.000				
*F* change	4.31, *p* =0.006					37.47, *p* =0.000					0.02, *p* =0.888				
*R* ^2^	0.07					0.34					0.34				
Adjusted *R*^2^	0.05					0.32					0.32				
*N*	189					189					189				

*The number of previous court hearings was measured on a five-point scale (1=0, 2=1, 3=2–10, 4=11–20, 5=more than 20)*.

**Table 12:** Regression results for procedural justice, outcome judgments, and their interaction on protest intentions.

Variable	Step 1				95% CI for *b*	Step 2				95% CI for *b*	Step 3				95% CI for *b*
	*b*	*β*	*t*	*p*		*b*	*β*	*t*	*p*		*b*	*β*	*t*	*p*	
Legal assistance (0=*no*, 1=*yes*)	0.16	0.04	0.48	0.630	−0.50, 0.82	−0.08	−0.02	−0.30	0.763	−0.61, 0.45	−0.09	−0.02	−0.32	0.751	−0.62, 0.45
Previous hearings	−0.03	−0.02	−0.20	0.842	−0.32, 0.26	−0.02	−0.01	−0.16	0.875	−0.25, 0.22	−0.02	−0.01	−0.15	0.882	−0.25, 0.22
Age	−0.25	−0.13	−1.71	0.088	−0.55, 0.04	−0.16	−0.08	−1.31	0.192	−0.39, 0.08	−0.15	−0.08	−1.26	0.210	−0.39, 0.09
Procedural justice						−0.31	−0.15	−2.09	0.038	−0.61, −0.02	−0.32	−0.16	−2.08	0.039	−0.62, −0.02
Outcome judgments						−1.01	−0.50	−7.02	0.000	−1.30, −0.73	−1.01	−0.50	−7.01	0.000	−1.30, −0.73
Outcome judgments×Procedural justice											−0.03	−0.01	−0.19	0.849	−0.29, 0.24
*df*	3					5					6				
*F*	1.21, *p* =0.308					22.47, *p* =0.000					18.63, *p* =0.000				
*F* change	1.21, *p* =0.308					53.33, *p* =0.000					0.04, *p* =0.849				
*R* ^2^	0.02					0.38					0.38				
Adjusted *R*^2^	0.00					0.36					0.36				
*N*	190					190					190				

*The number of previous court hearings was measured on a five-point scale (1=0, 2=1, 3=2–10, 4=11–20, 5=more than 20)*.

**Table 13:** Regression results for procedural justice, outcome judgments, and their interaction on self-esteem.

Variable	Step 1				95% CI for *b*	Step 2				95% CI for *b*	Step 3				95% CI for *b*
	*b*	*β*	*t*	*p*		*b*	*β*	*t*	*p*		*b*	*β*	*t*	*p*	
Legal assistance (0=*no*, 1=*yes*)	0.05	0.02	0.26	0.793	−0.31, 0.40	0.13	0.05	0.72	0.474	−0.22, 0.48	0.12	0.05	0.69	0.492	−0.23, 0.48
Previous hearings	−0.14	−0.13	−1.78	0.077	−0.30, 0.02	−0.14	−0.13	−1.74	0.083	−0.29, 0.02	−0.14	−0.13	−1.72	0.086	−0.29, 0.02
Age	0.03	0.03	0.39	0.696	−0.13, 0.19	0.01	0.01	0.18	0.857	−0.14, 0.17	0.02	0.02	0.22	0.826	−0.14, 0.18
Procedural justice						0.19	0.18	1.96	0.051	−0.00, 0.39	0.19	0.17	1.85	0.066	−0.01, 0.38
Outcome judgments						0.09	0.08	0.92	0.360	−0.10, 0.28	0.09	0.08	0.91	0.363	−0.10, 0.28
Outcome judgments×Procedural justice											−0.02	−0.02	−0.26	0.793	−0.20, 0.15
*df*	3					5					6				
*F*	1.06, *p* =0.366					2.72, *p* =0.022					2.26, *p* =0.039				
*F* change	1.06, *p* =0.366					5.12, *p* =0.007					0.07, *p* =0.793				
*R* ^2^	0.02					0.07					0.07				
Adjusted *R*^2^	0.00					0.04					0.04				
*N*	189					189					189				

*The number of previous court hearings was measured on a five-point scale (1=0, 2=1, 3=2–10, 4=11–20, 5=more than 20)*.

## Discussion

The present study critically examines the role of perceived procedural justice, and other important variables, in Dutch criminal court hearings. We think the message of what we learn from the reported findings is twofold. First, perceived procedural justice matters. That is, our findings showed robust associations between perceived procedural justice and trust in judges, outcome judgments, protest intentions, and state self-esteem. Second, processes of self-enhancement did not have the effects found by studies conducted in organizational contexts or laboratory settings. That is, outcome judgments and perceptions of everyday discrimination did not significantly moderate the associations between perceived procedural justice, on the one hand, and trust in judges, protest intentions, and state self-esteem on the other hand. In what follows, we deepen these conclusions. We then discuss the limitations of the present study, suggestions for future research that follow from these limitations, and practical implications of our findings.

### The Importance of Fair Procedures

An important finding of this study is that respondents who felt treated more fairly during their court hearings reported higher levels of trust in judges, judged their outcomes more positively, showed lower protest intentions, and displayed higher state self-esteem. These favorable reactions to perceived procedural justice indicate that, even in the real-life courtroom context of our study in which respondents risked actual sanctions, respondents cared not only about their outcomes but also about the way they were treated during their court hearings. Thus, the current study contributes to the ongoing academic debate on the relative importance of perceived procedural justice in real-life cases. Our findings support the argument by Casper et al. (1988) that the positive associations between perceived procedural justice and relevant other variables represent real-world phenomena that can also be observed outside the artificial settings of psychological laboratories, including criminal justice contexts (see also, for instance, Tyler, 1984, 1988, 2006; Paternoster et al., 1997; Tyler and Huo, 2002).

Our study also complements current insights into perceived procedural justice in criminal justice contexts by examining whether defendants’ reactions to perceived procedural fairness may be moderated by experiences of everyday discrimination and outcome judgments. Our results indicated that none of the interaction effects we examined were statistically significant. In other words, rather than being attenuated or reversed, the associations between perceived procedural justice and our other variables remained intact regardless of the extent to which respondents experienced discrimination in their daily lives and how positively or negatively they judged their outcomes. This might be interpreted as an indication of the robustness of the fair process effect. These findings also fit with other studies, which show that people belonging to ethnic minorities respond equally favorably to perceived procedural justice as do people from majority groups (Tyler, 2001; Sunshine and Tyler, 2003; Higgins and Jordan, 2005; Johnson et al., 2017).

### Different Cases and Contexts

We note that there may also be other possible explanations for the lack of statistically significant interaction effects in our study. These explanations may relate, for instance, to the type of cases examined and the context of our research. In the organizational and performance-oriented settings of previous studies examining attenuated or reversed fair process effects (e.g., Van den Bos et al., 1999; Brockner et al., 2009), negative outcomes were likely to threaten people’s self-esteem and thus make them look for opportunities to attribute these outcomes to external causes. In the courtroom context of the current study, negative case outcomes may not have posed a similar threat to respondents’ sense of self-worth. Hence, the lack of significant interaction effects might be explained by the legal context of our study. This indicates, we think, that more research is needed into the operations of self-enhancement processes in relevant legal contexts, such as criminal court hearings. Our study provides an important first step in this regard.

Similarly, the interaction between outcome judgments and perceived procedural justice has generally been found in work contexts or in other settings with different types of respondents than we examined in the current study (for overviews, see Brockner and Wiesenfeld, 1996; Brockner, 2010). Previous studies examining perceptions of actual defendants in criminal cases (Grootelaar and van den Bos, 2018) or undergraduates putting themselves in the position of defendants (Walker et al., 1974) did not find an interaction effect between outcomes and procedural justice. Our findings thus provide further support for the argument by Grootelaar and Van den Bos (2018), who did find interactions between perceived procedural justice and outcome favorability for motoring fine cases, that the type of case may play an important role in shaping people’s reactions to perceived procedural justice and outcome favorability in legal contexts.

### Conflicting Psychological Processes

Another potential explanation for not finding interactive effects of outcome judgments or perceived everyday discrimination and perceived procedural justice might be that conflicting psychological processes are at work. That is, the self-enhancement processes underlying attenuated or reversed fair process effects in other studies might play a role in the courtroom context of our study, but their effects may have been canceled out or overridden by other psychological processes (see also Brockner et al., 2009).

For instance, defendants who experience much discrimination in their daily lives may be pleasantly surprised by how fairly they feel treated during their court hearings, leading them to respond even *more* favorably to perceived procedural justice than defendants who experience little everyday discrimination. In the current paper, we explored whether defendants who experience much discrimination in their daily lives might respond *less* favorably to perceived procedural justice because of self-enhancement processes. These potential moderating effects of perceived everyday discrimination may have canceled each other out, resulting in non-significance of the interaction effect.

Defendants’ desire for fair treatment may also simply have overridden their self-enhancement motive. After all, perceived procedural justice may be desirable for various instrumental and non-instrumental reasons, as explained in the Introduction. These beneficial aspects of perceived procedural justice may have been stronger than defendants’ self-enhancement processes, resulting in favorable responses to procedures that defendants perceive as fair rather than unfair.

### Levels of Perceived Everyday Discrimination

Respondents’ relatively low levels of perceived everyday discrimination (*M*=2.48, SD=1.16, measured on a six-point scale) may be relevant as well. After all, respondents who scored one standard deviation above the mean level of perceived everyday discrimination (i.e., a score of 3.64) encountered negative treatment between a few times a year (score 3) and a few times a month (score 4). These experiences of discrimination may not have been sufficiently frequent to make defendants respond favorably to perceived procedural unfairness during their court hearings for self-enhancement reasons. Hence, we recommend that future studies examining these issues use samples in which levels of perceived everyday discrimination are likely to be higher.

### Limitations

An engaging aspect of our study, we think, is that we were able to study perceptions of actual defendants in single judge criminal cases after a 9-month period of data collection at the court of the mid-Netherlands. The flip side of this approach is that our sample is sufficiently large, yet smaller than we would have wanted ideally. For instance, a larger sample would have enabled us to robustly examine the three-way interaction between outcome judgments, perceived everyday discrimination, and perceived procedural justice. We would expect attenuated or reversed associations between perceived procedural justice and relevant other variables in particular among respondents with both relatively high levels of perceived everyday discrimination and relatively negative outcome judgments. Thus, future studies with larger samples are needed to better understand the issues examined in the current paper. Follow-up studies with larger samples could also examine, for instance, the possible role of being found guilty and seriousness of the sanction imposed. After all, more serious sanctions might pose a greater threat to defendants’ self-esteem, thereby making attenuated or reversed fair process effects more likely to occur.

We also note that we conducted our study at only one court and included only single judge criminal cases. Furthermore, the first author – who collected the bulk of the data – is a White and university-based researcher. As a result, interviewer effects may have played a role in our study. For instance, respondents may have concealed their levels of distrust in Dutch judges, as they may have considered the researcher as belonging to their outgroup (Hulst, 2017). Thus, we propose that it is important to replicate our study in other courts with different researchers and different types of court cases. Follow-up studies could also include defendants whom we were not able to include in our current sample, such as defendants in pre-trial detention.

In addition, the correlational design of this study does not allow for conclusions about any causal relationships between our variables. Thus, although we believe the field work element to be a strength of the paper, this also has methodological limitations. For instance, perceptions of procedural justice may influence defendants’ outcome judgments, and vice versa, which renders the analysis pertaining to Hypothesis 3 difficult to interpret.

Furthermore, a possibility that cannot be ruled out in our correlational design is that some of the effects that we examined with this hypothesis might already be present in the variation in the independent variables, making the validity of the interaction between the two terms difficult to assess. For example, after having received a negative case outcome, some defendants may have re-evaluated their sense of procedural fairness because of their need for self-enhancement. Thus, part of what might be going on in our analyses could be motivated reasoning regarding procedural fairness once defendants received a negative case outcome. This would fit with findings reported by Lilly and Wipawayangkool (2018) obtained in a non-courtroom setting. They found that external self-serving bias and self-threat following unfavorable outcomes were negatively related to procedural justice perceptions. Thus, studies using experimental control can clarify issues of causality and are therefore a viable avenue for future research into the issues examined here.

### Practical Implications

While recognizing these limitations, we think the findings of our study can have some important practical implications. Trust in judges, for instance, is an issue that has the Dutch judiciary’s ongoing attention. Although the level of trust in the Dutch judiciary is relatively high compared to trust in other Dutch governmental institutions and judiciaries in other European countries (Ridder et al., 2019; Bovens, 2020), safeguarding this trust is considered important (Grimmelikhuijsen, 2018). Fair procedures, in terms of objective legal standards as well as people’s subjective perceptions, can play an important role in this regard. This is relevant not only with a view to maintaining and possibly increasing trust in judges as an end in itself, but also because trust in judges is related to other important attitudes and behaviors, such as perceived legitimacy and compliance with the law (Grootelaar and van den Bos, 2018).

Our finding that perceived procedural justice is negatively associated with protest intentions can be of interest to legal policymakers and judges as well. Although reporting protest intentions is not the same as actually appealing a verdict, the two are likely to be related. It is noteworthy in this regard that more than 90% of appeals to criminal verdicts are initiated by defendants (Croes, 2016). Promoting procedural justice could therefore be a way to decrease the number of appeals and the social costs associated therewith. These social costs may concern not only financial costs but also costs in terms of quality of adjudication, as judges’ workload is considered a threat for impartial adjudication by one out of five Dutch judges (Weijers, 2019). Taken together, we think our findings regarding the importance of procedural justice in the criminal courtroom context are relevant for both their contribution to procedural justice theory and their possible implications for legal practice.

## Data Availability Statement

The datasets presented in this study can be found in online repositories. The names of the repository/repositories and accession number(s) can be found at: https://doi.org/10.24416/UU01-74OU94.

## Ethics Statement

The studies involving human participants were reviewed and approved by the ethical assessment committee of the faculty of Law, Economics, and Governance at Utrecht University. Written informed consent for participation was not required for this study in accordance with the national legislation and the institutional requirements.

## Author Contributions

LA designed the study, including the questionnaire and the study’s procedures, organized approval by the court, organized and liaised about ongoing data collection within the court, collected most of the data and directed data collection by a research assistant, analyzed the data, interpreted results, and wrote the manuscript. KB provided conceptualization and theoretical input and aided in designing the study and questionnaire, provided conceptualization and theory used to integrate findings, co-interpreted results, and edited the manuscript. EM provided input for the design and setup of the study and commented on several drafts of the manuscript, including editing of the manuscript. All authors contributed to the article and approved the submitted version.

## Funding

This research was funded by a Research Talent grant awarded by the Dutch Research Council (NWO, 406.16.558).

## Conflict of Interest

The authors declare that the research was conducted in the absence of any commercial or financial relationships that could be construed as a potential conflict of interest.

## Publisher’s Note

All claims expressed in this article are solely those of the authors and do not necessarily represent those of their affiliated organizations, or those of the publisher, the editors and the reviewers. Any product that may be evaluated in this article, or claim that may be made by its manufacturer, is not guaranteed or endorsed by the publisher.

## Footnotes

^1^Previous research suggests that criminal defendants sometimes find the term “fair” easier to understand and use than “just” and has therefore treated these terms as synonyms (Ansems et al., 2020). Given the similarity in research context, the current paper uses the terms “justice” and “fairness” interchangeably as well.

^2^In this paper, a “non-Western ethnic-cultural background” refers to being born in a non-Western country, which according to Statistics Netherlands (2018) refers to countries in Africa, Latin America, and Asia (excluding Indonesia and Japan), or Turkey. We also use the term to refer to persons whose parents or other ancestors were born in a non-Western country. We included these latter respondents in our sample because they, too, might experience discrimination in their daily lives due to their ethnic-cultural background.

^3^In this paper, we focus on the hypotheses that are central to the line of reasoning presented here. We also performed a small number of additional analyses. For example, we examined whether there was a significant three-way interaction between outcome judgments, perceived everyday discrimination, and perceived procedural justice. This analysis was conducted for exploratory purposes only, however, and is not reported in the current paper. After all, a power analysis (Faul et al., 2007) showed that, to achieve sufficient statistical power of 0.80 (Cohen et al., 2003) to detect the three-way interaction, with *Î±* = 0.05 and a relatively small effect size (*f*^2^ = 0.02), at least 387 respondents were needed. Complete details and results are available with the first author on request.

^4^Like some other Dutch courts, the court of the mid-Netherlands has buildings in multiple cities (including Utrecht, Lelystad, and Almere).

^5^After having signed a confidentiality agreement, at the beginning of each week of data collection the first author received an overview of single judge criminal cases that would be heard that week, detailing times, charges, and defendants’ names. We used these overviews to decide in which hallway to wait for potential respondents when there were multiple court hearings taking place at different floors at the same time (to be able to approach as many eligible defendants as possible) and we shredded these overviews at the end of each week for reasons of confidentiality.

^6^Because we report all measures used in our questionnaire, we note that we also measured respondents’ external attribution ratings and the extent to which they identified with their ethnic-cultural subgroup. The items we used to measure respondents’ external attribution ratings yielded a very low Cronbach’s alpha (*Î±* = 0.17), rendering this variable unsuitable for analysis. We included the items on subgroup identification in our questionnaire for potential additional analyses, as previous work suggests that attributions to discrimination might not protect the self-esteem of people who strongly identify with their ethnic-cultural subgroup (McCoy and Major, 2003). In the end, we did not perform these analyses because of power issues. Hence, we decided to drop these variables from the current paper. Complete details and results are available on request.

^7^We conducted all analyses involving perceived procedural justice with both this six-item scale and the entire 17-item scale. In the Results section, we explicitly note when these analyses yielded (slightly) different results with regard to our main findings.

^8^There were missing values for perceived ground of discrimination (51 missing values), trust in judges (one missing value), grade for trust in judges (12 missing values), and self-esteem (one missing value). There were also some missing values for gender (one missing value), age (three missing values), highest completed level of education (eight missing values), offense (eight missing values), type of verdict (six missing values), sanction received (32 missing values), legal assistance (four missing values), and number of previous court hearings (one missing value).
